# An Enhanced Robot Massage System in Smart Homes Using Force Sensing and a Dynamic Movement Primitive

**DOI:** 10.3389/fnbot.2020.00030

**Published:** 2020-06-29

**Authors:** Chunxu Li, Ashraf Fahmy, Shaoxiang Li, Johann Sienz

**Affiliations:** ^1^Centre for Robotics and Neural Systems, University of Plymouth, Plymouth, United Kingdom; ^2^Shandong Marine Corrosion and Safety Protection Engineering Research Center, Qingdao University of Science and Technology, Qingdao, China; ^3^ASTUTE 2020 in Future Manufacturing Research Institute, College of Engineering, Swansea University, Swansea, United Kingdom; ^4^Department of Electrical Power and Machines, Helwan University, Helwan, Egypt

**Keywords:** hybrid force/position, teaching by demonstration, dynamic motion primitive, dynamic time warping, gaussian mixture regression

## Abstract

With requirements to improve life quality, smart homes, and healthcare have gradually become a future lifestyle. In particular, service robots with human behavioral sensing for private or personal use in the home have attracted a lot of research attention thanks to their advantages in relieving high labor costs and the fatigue of human assistance. In this paper, a novel force-sensing- and robotic learning algorithm-based teaching interface for robot massaging has been proposed. For the teaching purposes, a human operator physically holds the end-effector of the robot to perform the demonstration. At this stage, the end position data are outputted and sent to be segmented via the Finite Difference (FD) method. A Dynamic Movement Primitive (DMP) is utilized to model and generalize the human-like movements. In order to learn from multiple demonstrations, Dynamic Time Warping (DTW) is used for the preprocessing of the data recorded on the robot platform, and a Gaussian Mixture Model (GMM) is employed for the evaluation of DMP to generate multiple patterns after the completion of the teaching process. After that, a Gaussian Mixture Regression (GMR) algorithm is applied to generate a synthesized trajectory to minimize position errors. Then a hybrid position/force controller is integrated to track the desired trajectory in the task space while considering the safety of human-robot interaction. The validation of our proposed method has been performed and proved by conducting massage tasks on a KUKA LBR iiwa robot platform.

## 1. Introduction

With the continuous development of technology, many traditional industries have been gradually replaced by high-tech products. Among them, the development of robot technology plays an important role. Robot technology integrates multiple disciplines, such as machinery, information, materials, intelligent control, and biomedicine, and intelligent service robots are intelligent equipment that provide humans with necessary services in an unstructured environment. The tasks that a robot can accomplish are divided into two categories. One is non-contact operation: the robot carries and operates the target in a free space, and the simple position control can be used for another type of contact operation. For tasks such as assembly, grinding, polishing, debarring, etc., simple position control is no longer sufficient. In such cases where the contact force is required, the slight positional deviation of the robot end may cause the contact force to damage the robot and the target. Therefore, the contact force control function must be added to the contact robot control system. The traditional robot control system for free space motion cannot meet the requirements of the extended application of robots. The perception and control of the robots of environmental forces are problems that current robot technology needs to solve. The control strategy combined with the end pose and the contact force for a robot is defined as the robotic force/position control. The key technologies of the current robotic force/position control mainly include the following aspects:

1. the robotic impedance control under the condition that the environmental parameter geometry and dynamic model parameters are unknown or changed;
2. the robotic force/position hybrid control under system interference (model error, measurement noise, and external input interference);
3. the robotic collision contact control;
4. the engineering implementation of robotic force/position control.

**Figure 1:**
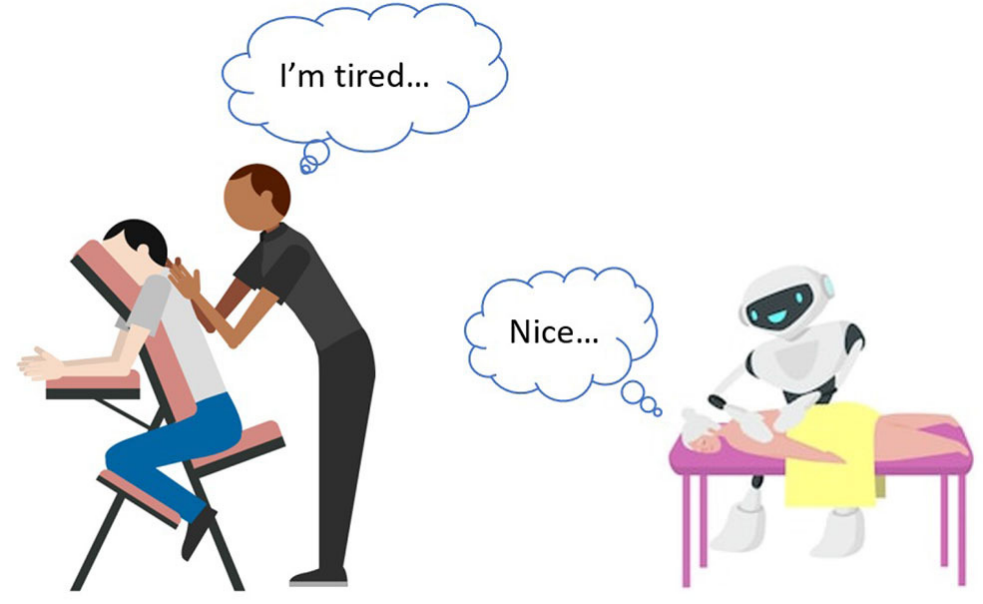
Conception of robot massaging in smart homes.

Service robots mimic humans in shape and behavior design; for instance, they have hands, feet, heads, and a torso. This helps them to adapt to human life and a work environment. Service robots replace human beings to complete various tasks and expand human capabilities in many aspects. Recently, research into robot massaging in smart homes (shown in Figure 1) has attracted widespread attention. Massaging is an important part of people's realization of a healthy lifestyle that can relieve stress and relax the body. By relaxing the muscles and joints, massages improve the flexibility of the body's joints and muscles, thereby reducing muscle pain caused by poor posture. However, for the manual massage tasks, it takes lots of energy and labor cost. Thus, in recent years, robot massaging has become a research hotspot in the field of robots. In 1996, the Mechatronics Research Center of Japan's Sanyo Electric Co., Ltd. designed a mechanical therapy unit and verified the feasibility of developing an intelligent massage robot (Kume et al., 1996). In 2000s, Toyohashi University of Technology and Japan's Gifu Institute of Technology carried out research on humanoid multi-finger massage robots with four fingers and 13 joints (Zhang and Zhang, 2017). Since 2004, Chinese scholars have carried out research on massage robots based on the theory of traditional Chinese massage and systematically discussed the robotic synthesis of various traditional Chinese medical massage techniques (Ma et al., 2005). However, existing massage robots are complicated to operate with limited functions, a large size with bulky equipment, and they are also expensive. Moreover, most of the existing robot massage technology depends on the function of the robot product itself; in other words, these robots cannot be used for operations other than massage, and their tandem structure stiffness is large, the motion inertia is unstable, and the working space and flexibility are limited (Zhang and Zhang, 2017; Field, 2018). To overcome the abovementioned issues, this paper develops a teaching-by-demonstration-based interface using a hybrid position/force control strategy with adjustable stiffness, which can be implemented onto a general robot manipulator with high accuracy, taught by a human operator.

Goradia et al. (2002) studied robotic forces/positions under different surfaces (i.e., unknown to the environment) to better facilitate the polishing or grinding processes. Scherillo et al. (2003) studied the force/position control of multi-finger grasping, Nguyen et al. (2000) used the sliding mode variable structure to control the force and the position of the robot, Ha et al. (2000) and used the impedance control method to control the force/position of the robot. In Fanaei and Farrokhi (2006), authors used a robust adaptive method to control the force and the position of the robot. Research on the force/position intelligent control of the robot is still mainly theoretical, the technical realization is at the stage of exploration, and there is still a certain distance to go in terms of promotion and practicality.

Movement is necessary to directly produce skill effects. The motion model generates a continuous robot space state representation skill offline or online with clear meanings related to the physical system, such as position, attitude, and contact force. Generally, the motion is assumed to be nonlinear. The motion model mainly includes two categories: trajectory encoding modeling and dynamic system modeling. Trajectory encoding is a compact mathematical model that represents the shape, constraints, and other information of the trajectory. When the human operators teach and store one or more specific fixed trajectories in the robot, then the robot can accurately reproduce the trajectory when the motion skills are executed. Calinon and Billard (2007) used trajectory encoding with a multivariate Gaussian mixture model (GMM), which expresses the trajectory in a fixed coordinate system. However, considering reality, the trajectory often needs to be expressed in different reference coordinate systems, so the transformation relationship of the reference coordinate system may also change. Thus, in Silvério et al. (2015), they proposed a task-parameterized Gaussian Mixture Model (TP-GMM) wherein the origin and rotation transformation matrix of the coordinate system are used as the task parameters in the model, which allows the observation and reproduction of the motion trajectory in different reference coordinate systems. However, the trajectory encoding modeling method only explicitly generates a fixed trajectory. When the robot is disturbed and deviates from the trajectory, the trajectory cannot be adjusted in real time, and a new trajectory needs to be regenerated.

**Figure 2:**
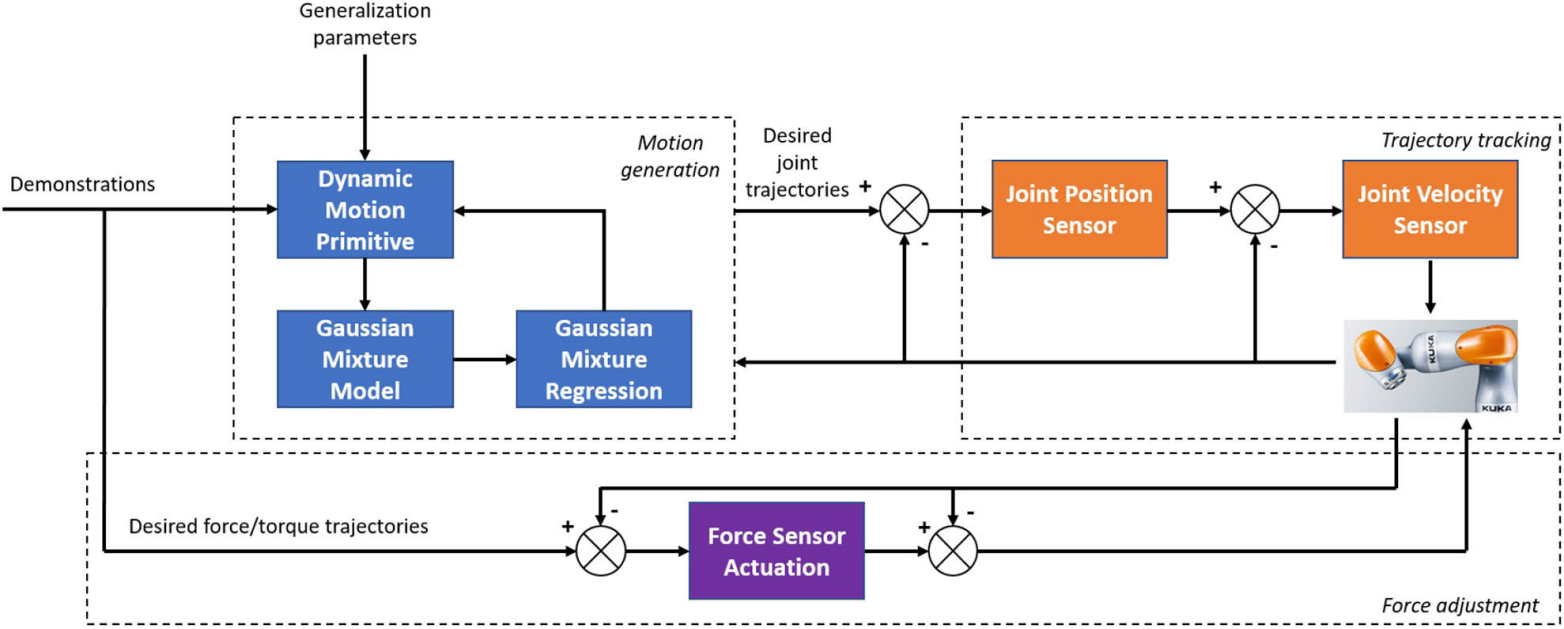
Diagram of the proposed control architecture.

The dynamic system is automatically evolved according to certain rules. Compared with the trajectory encoding, there are two main differences: the dynamic system does not explicitly depend on the time variable–only the relationship between the spatial state and its time derivative; the dynamic system can be online. The trajectory generated by such a dynamic system obtains the online adaptive ability for disturbance. DMP was proposed by Schaal (2003) in 2002 as a dynamic system model that can generate trajectories of arbitrary shapes where its basic idea is to drive a transform system with a canonical system. Nanayakkara et al. (2004) proposed a Mixture of motor primitives (MoMP) using a Gaiting network to calculate the weight of each DMP in the current state. Ideally, the Gaiting network should only choose one DMP implementation. However, the reality is more complicated; in order to achieve a good generalized performance, MoMP weights the output of all DMPs based on the weight of the output of the gated network to obtain the final output. In Matsubara et al. (2011), the coordination matrix is used to represent the coupling relationship between multiple DMPs corresponding to multiple degrees of freedom, and the iterative dimensionality reduction method is used to reduce the unnecessary degrees of freedom in the cooperation matrix, which is beneficial to enhancing the learning efficiently. DMP mainly depends on the target state and weight coefficient. The former is determined by the environment or setting. The latter can learn from the teaching trajectory based on the linear weighted regression (LWR) method. However, the LWR can only learn the DMP model parameters from a single demonstration. This paper mainly presents two aspects of research: the theory of hybrid force/position control with direct human–robot interaction and the experimental studies on a real robotic platform. The motion planning is performed in 3D task space, where the GMM is employed to evaluate the DMP to learn from multiple demonstrations, and GMR is used for the reproduction of the generalized trajectory with a smaller error. For the force input aspects, a hybrid force/position controller is introduced to ensure the safety of direct human–robot interaction. An overview of the presented control architecture is shown in Figure 2. The contributions of this paper are summarized.

1. This paper employs a teaching interface to perform the robot massaging under the demonstrations of the human operator, and the experimental studies show that, after the teaching process, the robot can generate an even smoother trajectory that strictly adheres to what is requested to be followed.
2. The application of the hybrid force/position control scheme takes care of the safety issues and achieves massage services performing without knowing subject profiles.
3. The generalization functions of our proposed method supplies a more flexible and convenient option with only once teaching for multiple tasks to the carers and patients, which promotes the user experiences.

## 2. Data Preprocessing

### 2.1. Motion Skills Segmentation

FD approximates the derivative by the limitable variance and finds an estimated solution for the differential equation (Chelikowsky et al., 1994). The differential form, especially, is to substitute the differential with a finite difference and the derivative with a finite differential quotient so as to roughly change the fundamental formula and limit state (usually the differential equation) into the differential equation (Algebraic equation) (Gear, 1988). The solution of the differential equations problem is updated so that the algebraic equations problem is resolved.

Segmentation of skills is usually a complicated and systematic process that requires more time and efforts on its algorithms designing. For such a complex method, there are often difficulties in setting the priori parameters. Considering the situation that the massaging motion is planed on a horizontal plane, and the force is applied into vertical direction of that plane, during the massaging tasks, it is easy to track for the system at which point the position being massaged has been changed. This is because its changing position represents the value varying in *z* coordinates. Consequently, a simple segmentation method (FD) is employed in this paper. In view of *y*_*i*_=*f*(*z*_*i*_) relies upon the *z*_*i*_ variables, where *z*_*i*_ denotes the coordinate values in the vertical direction at the *i*_*th*_ time series, and *y*_*i*_ represents the corresponding spatial sequence of the whole dataset. If *z*_*i*_ is changed into *z*_*i*+1_, the corresponding changes in the whole dataset is *df*(*z*_*i*_) = *f*(*z*_*i*+1_) − *f*(*z*_*i*_) and *d* is the differentiation operator. Difference has a differential-like arithmetic value. This displays the following equation (Li et al., 2018):

(1) $$\begin{array}{l} f^{\prime }\left(z_{i}\right)=df\left(z_{i}\right)=y_{i}^{\prime }\approx \frac{y_{i+1}-y_{i}}{z_{i+1}-z_{i}} \end{array}$$

where one significant massaging aspect is that the endpoint would be lifted once every single massaging task is done. The “*z*” alignment values of the experimental data are thus viewed as the segmentation reference. We have received, pursuing the FD,

(2) $$\begin{array}{l} \xi \left(y_{i}^{\prime }\right)=sign\left(|y_{i}^{\prime }|-\theta \right) \end{array}$$

Where ξ is the gaping variable, abd θ is a constant. We could modify the segmented characters, such as, θ, here θ=0.5, by giving different values of θ. Sign is the Signum function, and, for each component of ξ, the formulation could be described as following:

(3) $$\begin{array}{l} sign\left(\xi \right)=\{\begin{array}{cc} -1 & if\;\xi <0,\\ 0 & if\;\xi =0,\\ 1 & if\;\xi >0. \end{array} \end{array}$$

**Figure 3:**
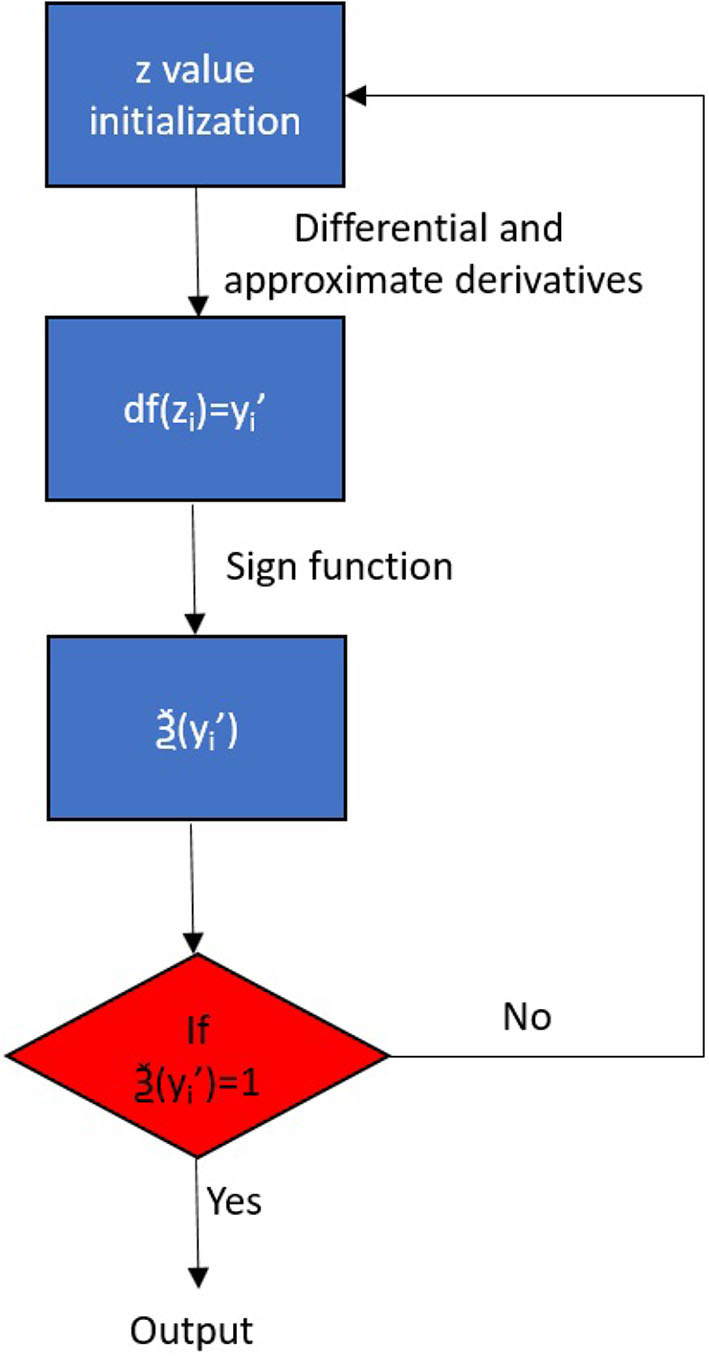
The flowchart of the segmentation.

Up to now, the segmented motion trajectories *sign*(ξ) for massaging have been outputted to different local text files for the use of GMM and DMP generalization, which correspond to the “*z*” coordinate information in the robot space, suddenly and sharply rising, which implies every time the massaging for one position was done. The flowchart of the segmentation is shown in Figure 3.

### 2.2. Alignment of Time Series

The DTW employed in this paper aligned the outputted trajectories curves in the form of *W* = {*w*_1_, *w*_2_, ⋯ , *w*_*p*_, ⋯ , *w*(*p*)} in which *w*(*p*) = (*i*_*p*_, *j*_*p*_) denotes the match variable in Petitjean et al. (2014). In this situation, the skewed characteristic W is required to reduce the discrepancy between the pending trajectory and the reference trajectory. The formula is therefore described as

(4) $$\begin{array}{l} D=min\overset{K}{\underset{k=1}{\sum }}d\left[w\left(p\right)\right] \end{array}$$

where *d*[*w*(*p*)] = *d*[*T*_*i*_(*p*), *R*_*j*_(*p*)] represents the measured distance from the *i*(*p*)th featured point of the pending trajectory to the *j*(*p*)th featured point of the reference trajectory, which is normally described by a square measure specified as follows:

(5) $$\begin{array}{l} d\left[w\left(p\right)\right]={\left[T_{i}\left(p\right)-R_{j}\left(p\right)\right]}^{2} \end{array}$$

We need to construct a matrix grid of *m* × *n* in order to coordinate the two specimens; the matrix element (*I, j*) represents the range *d*(*T*_*i*_, *R*_*j*_) between the two points *T*_*i*_ and *R*_*j*_, and each matrix element (*i, j*) represents the alignment of points *R*_*j*_ and *R*_*j*_. DTW aims to find a direction which reflects the matched points for both samples to be determined via several grid points. First we illustrate, as *D*_*Acc*_(*i, j*), the total minimum range between the two trajectories, then we consider (Senin, 2008)

(6) $$\begin{array}{l} D_{Acc}\left(i,\;j\right)=d\left(T_{i},\;R_{j}\right)+min_{\left(q_{i},\;q_{j}\right)}\left[D_{Acc}\left(q_{i},\;q_{j}\right)\right] \end{array}$$

where (*q*_*i*_, *q*_*j*_) belongs to the set of points between (1, 1) and (*i, j*) within a certain direction. From the above components it can be seen that the average cumulative distance of the (*i, j*) element is linked not only to the regional distance *d*(*T*_*i*_, *R*_*j*_) of the own values *T*_*i*_, *R*_*j*_ but also to the total cumulative distance earlier than this stage in the coordinate system.

We thus assume that (*i, j* − 1), (*i* − 1, *j*), and (*i* − 1, *j* − 1) for any point *c*(*p*) = (*i, j*) within the coordinate system may enter the preceding point of *c*(*p*), so the choice of the preceding variable also needs to agree with the above three factors. We may measure the corresponding DTW range between the test pattern vector and the comparison model vector, as shown below, according to the formula.

(7) $$\begin{array}{l} D^{\prime }=D_{Acc}\left(L_{1},\;L_{2}\right). \end{array}$$

## 3. Trajectory Generation

DMP is an innovative model (Schaal, 2006) dynamic system learned from biological research that learns from motor primitives. The definition of dynamic primitive can be separated into two groups. One is to use different formulas based on dynamic system to represent the state, and the other is to produce the track by interpolation through the interpolation points (Li et al., 2017). DMP is made up of two parts: the transformed model *r* and the canonical framework *h*. The equation is shown as follows:

(8) $$\begin{array}{l} ṡ=h\left(s\right) \end{array}$$

(9) $$\begin{array}{l} ṫ=r\left(t,\;s,\;w\right) \end{array}$$

where *t* and *s* are the transformed process states and the canonical function, and the canonical system output variable *h* is referred to as *w*.

The canonical model is defined by an exponential differential equation, which is given:

(10) $$\begin{array}{l} \tau ṡ=-\alpha_{f}s \end{array}$$

where *s* is a step function varying from 0 to 1, τ > 0, and α_*f*_ is a temporal scaling variable and a balanced component.

The transformed model consists of two nonlinear term sections and a Cartesian space spring damping mechanism, the formulas are defined as (Schaal, 2006):

(11) $$\begin{array}{l} \tau \dot{v}=k\left(g-p\right)-cv+X\left(g-p_{0}\right) \end{array}$$

(12) $$\begin{array}{l} \tau ṗ=v \end{array}$$

where *p*_0_ is the starting position, *p* ∈ *R* is the Cartesian position, *v* ∈ *R* is the end-effector velocity of the robot, *g* is the goal, *k* is the spring variable, and *c* is the damping factor. *X* is a conversion method of dynamic nonlinear structures capable of transforming the outcomes of the canonical model found in the following formula:

(13) $$\begin{array}{l} X=\overset{N}{\underset{i=1}{\sum }}w_{i}l\left(s\right) \end{array}$$

Where the GMM number is *N*, *w*_*i*_ ∈ *R* is the weights, and *l* is the uniform radial variable value that can be supplied as follows:

(14) $$\begin{array}{l} l\left(s\right)=\frac{exp\left(-h_{i}{\left(s-c_{i}\right)}^{2}\right)}{\sum_{m=1}^{N}exp\left(-h_{m}{\left(s-c_{m}\right)}^{2}\right)} \end{array}$$

where *c*_*i*_ > 0 are the centers, and *h*_*i*_ > 0 are the widths of the functions of the Gaussian foundation. *N* is the number of functions in Gaussian.

In addition, we can use the weight variable to produce motions by specifying the starting point of the canonical process (*s*=0) *X*_0_ and aim *g*, which is the canonical system integration. The theory of DMP is to measure the nonlinear transition feature *X* by observing the presenter's movements. Nonetheless, there are drawbacks in developing a multi-demonstration conversion model, which is why the GMM is used to solve the above problems.

GMM's parameter estimation is the method by which the design parameters are obtained under certain conditions. In fact, it is the process of knowing the parameters of the model, namely, the method of resolving $\lambda =\left\{\mu_{i},\underset{i}{\sum },\omega_{i}\right\}$ to bring the GMM sequence of observation (Tong and Huang, 2008). The most commonly employed parameter estimation is the total probability approximation process. The basic idea is to consider the system parameter λ when the peak likelihood of GMM is obtained by providing the observation sequence X obtained by DMP from the previous chapter, then λ is the model. The optimum function, λ, defines to the maximum extent practicable the distribution of the observed string.

The end goal of the total probability calculation after providing the training information is to seek a template variable that maximizes the GMM's likelihood. For a training vector series of *X* = {*x*_1_, *x*_2_…*x*_*D*_} of duration D, it is possible to describe the likelihood of GMM as

(15) $$\begin{array}{l} P\left(X|\lambda \right)=\overset{D}{\underset{t=1}{\prod }}P\left(X_{t}|\lambda \right) \end{array}$$

The parameter λ is then continuously updated until a set of parameters λ is found to maximize *P*(*X* ∣ λ):

(16) $$\begin{array}{l} \hat{\lambda }=arg\underset{\lambda }{max}P\left(X|\lambda \right) \end{array}$$

For the convenience of analysis, *P*(*X* ∣ λ) usually takes its log likelihood, giving us

(17) $$\begin{array}{l} log\left(P\left(X|\lambda \right)\right)=\overset{D}{\underset{t=1}{\sum }}logP\left(X_{t}|\lambda \right) \end{array}$$

The Expectation Maximization (EM) algorithm can be used for parameter estimation provided that there is a relatively complex nonlinear interaction between the probability function and the template parameters, and the peak value cannot be determined according to the standard probability estimation process. The EM algorithm is in essence an iterative method for calculating the probability model's maximum likelihood. The process of each iteration is to estimate the unknown data distribution based on the parameters that have been acquired and then calculate the new model parameters under the maximum likelihood condition. Let the initial model parameter be λ, which satisfies

(18) $$\begin{array}{l} P\left(X|\lambda^{\prime }\right)⩾P\left(X|\lambda \right) \end{array}$$

Next, we calculate the new model variable λ′ according to the equation above and then use the λ′ parameter as the original parameter for the next iteration. It iteratively iterates until the state of convergence is met. Here we assume a Q function that represents the E phase of the EM process shown below:

(19) $$\begin{array}{l} Q\left(\lambda ,\;\lambda^{\prime }\right)=\overset{M}{\underset{i=1}{\sum }}P\left(X,\;i|\lambda \right)logP\left(X,\;i|\lambda \right) \end{array}$$

where *i* is an elusive and unpredictable secret country. *Q*(λ, λ′) corresponds to all observable data's log likelihood assumptions. Calculating the maximum value of *Q*(λ, λ′) increasing give the maximum log likelihood of the observed data, which is the M stage of the EM process. It is possible to obtain replacement formulas (15) and (16) for equations (6):

(20) $$\begin{array}{l} Q\left(\lambda ,\;\lambda^{\prime }\right)=\overset{M}{\underset{i=1}{\sum }}\overset{T}{\underset{t=1}{\sum }}r_{t}\left(i\right)log\omega_{i}b_{i}\left(x\right) \end{array}$$

(21) $$\begin{array}{l} r_{t}\left(i\right)=P\left(X_{t},\;i|\lambda \right) \end{array}$$

The approximate values of each variable are then computed according to E and M. Phase E calculates the posterior likelihood of the *t*_*th*_ test *X*_*t*_ of the training data according to the Bayesian equation in the *i*_*th*_ state; phase M first uses the Q method to extract the three parameters independently and then evaluate the corresponding figures. To re-evaluate the variables, we iteratively perform measures E and M. The loop is halted when the peak value of the likelihood function is reached.

The first step when using the EM method to calculate the GMM parameters is to determine the number of Gaussian components in the GMM, such as system M order and model initial parameter λ (Howlett et al., 2009). Based on the actual situation, such as the sum of training data, it is appropriate to choose the order M of the template. The most widely employed approach for the model's initial variable λ is the K-means algorithm. Currently, the K-means algorithm is the simplest and most effective classification algorithm, commonly used in different models (Nazeer and Sebastian, 2009). The GMM used in this paper chooses the basic parameters using the K-means method. The K-means algorithm partitions the information into K clusters according to the in-cluster number of squares in the category theory. After using the K-means method to cluster the feature vectors, the mean and variance of each group are determined and the percentage of each class 'feature vectors is calculated as the blending weight (Tatiraju and Mehta, 2008). The average, variance, and combined weight can be then collected as the predicted values. Finally, by applying the GMR, the same theory as our previous work (Li et al., 2018), the reconstructed motion trajectories can be then obtained.

## 4. Hybrid Force/Position Control

The robot kinematics model of n DOF robot is presented in the subsequent form

(22) $$\begin{array}{l} x\left(t\right)=\text{Ψ}\left(\Theta \right) \end{array}$$

where x(t) ∈ R^*n*^ represents the position and direction vector, and Θ ∈ R^*n*^ represents the joint angle vector. The inverse kinematics are

(23) $$\begin{array}{l} \Theta \left(t\right)={\text{Ψ}}^{-1}\left(x\right). \end{array}$$

The derivative of (23) can thus be rewritten:

(24) $$\begin{array}{l} ẋ\left(t\right)=J\left(\Theta \right)\dot{\Theta } \end{array}$$

where *J*(Θ) is the Jacobian matrix of the robot. Moreover, differentiating (24), we can get

(25) $$\begin{array}{l} ẍ\left(t\right)=\dot{J}\left(\Theta \right)\dot{\Theta }+J\left(\Theta \right)\ddot{\Theta }. \end{array}$$

The relationship between wrench and joint force can be described:

(26) $$\begin{array}{l} T_{ext}=J^{\text{T}}\left(\Theta \right)f. \end{array}$$

In addition, the robot manipulator dynamics in joint space is

(27) $$\begin{array}{l} M_{\Theta }\left(\Theta \right)\ddot{\Theta }+C_{\Theta }\left(\Theta ,\dot{\Theta }\right)\dot{\Theta }+G_{\Theta }\left(\Theta \right)+T_{fric}=T+T_{ext} \end{array}$$

where $\overset{∙}{\Theta }$ and $\ddot{\Theta }$ are the vectors of velocity and accelerations, respectively. *Mq*(Θ) ∈ R^*n*^ is the inertia matrix; $C_{\Theta }(\Theta ,\overset{∙}{\Theta }$) is the Coriolis and centripetal torque; *G*_Θ_(Θ) is the gravity; *T* is the robot torque; *T*_*fric*_ is the friction torque and *T*_*ext*_ is the external torque.

- *Property 1*: Matrix *M*_Θ_(Θ) is bounded above and below and positive definite symmetric.
- *Property 2*: Matrix $M_{\Theta }\left(\Theta \right)\ddot{\Theta }-2C_{\Theta }\left(\Theta ,\overset{∙}{\Theta }\right)$ is skew symmetric. matrix.

A force-position model of the relationship between the external force and position in joint space is

(28) $$\begin{array}{r} D_{g}J\left(\Theta \right)\left({\ddot{\Theta }}_{r}-|{\ddot{\Theta }}_{g}\right)+\left(C_{g}J\left(\Theta \right)+M_{g}\dot{J}\left(\Theta \right)\right)\left({\dot{\Theta }}_{r}-{\dot{\Theta }}_{g}\right)\;\\ +K_{g}\left(\phi \left(q_{r}\right)-\phi \left(q_{g}\right)\right)=-J^{-T}T_{ext} \end{array}$$

where $q_{g}\in {\text{R}}^{n}$ and $q_{r}\in {\text{R}}^{n}$ are the desired trajectory generated from GMR algorithm and virtual desired trajectory, respectively. The *M*_*g*_, *C*_*g*_, and *K*_*g*_ are gain matrix of the mass, damping and stiffness matrices designed by the controller, respectively.

- *Assumption 1*: *c*_1_; *c*_2_; and *c*_3_ are positive constants, and both *q*_*g*_ and *q*_*r*_ are differentiable and bounded: $q_{g},q_{r}\leq c_{1},{\overset{∙}{\Theta }}_{g},{\overset{∙}{\Theta }}_{r}\leq c_{2},{\ddot{\Theta }}_{g},{\ddot{\Theta }}_{r}\leq c_{3}$.
- *Remark 1*: In the specific cases, force-position models such as the damping-stiffness model and stiffness model are applied

(29) $$\begin{array}{r} C_{g}\left({\dot{\Theta }}_{r}-{\dot{\Theta }}_{g}\right)+K_{g}\left(q_{r}-q_{g}\right)=-T_{ext}\\ K_{g}\left(q_{r}-q_{g}\right)=-T_{ext} \end{array}$$

In the case that the desired manipulator's motion is free and no external collisions are generated, we can get *q*_*r*_ = *q*_*g*_; *T*_*ext*_ = 0. In the opposite, while there is an external collision, the robot will generate and follow the new trajectory, which is the adaptation to the force-position model and the external torque specified in Bernhardt et al. (2005) illustrates the relationship.

Regarding to the safety consideration, a moveable limit has been added to the KUKA iiwa platform in both of the Cartesian spaces to make sure the manipulator can only reach the areas in front of it with a radian of 0.6 m; in this case, the robot manipulator cannot fully stretch. Meanwhile, the stiffness of its endpoint in all directions is set upon a reasonable level, hence participants can easily afford the force from the robot. Furthermore, by adjusting the threshold in **SafetyConfiguration.sconf** of the KUKA controller, the Collision Detection Framework can be activated. It is relevant to decide at what levels the external force is to lock down the robot to protect the participants. Here, we set up this parameter at a low level, which avoids all the touching with high force.

## 5. Experimental Studies

### 5.1. Experiment Setup

**Figure 4:**
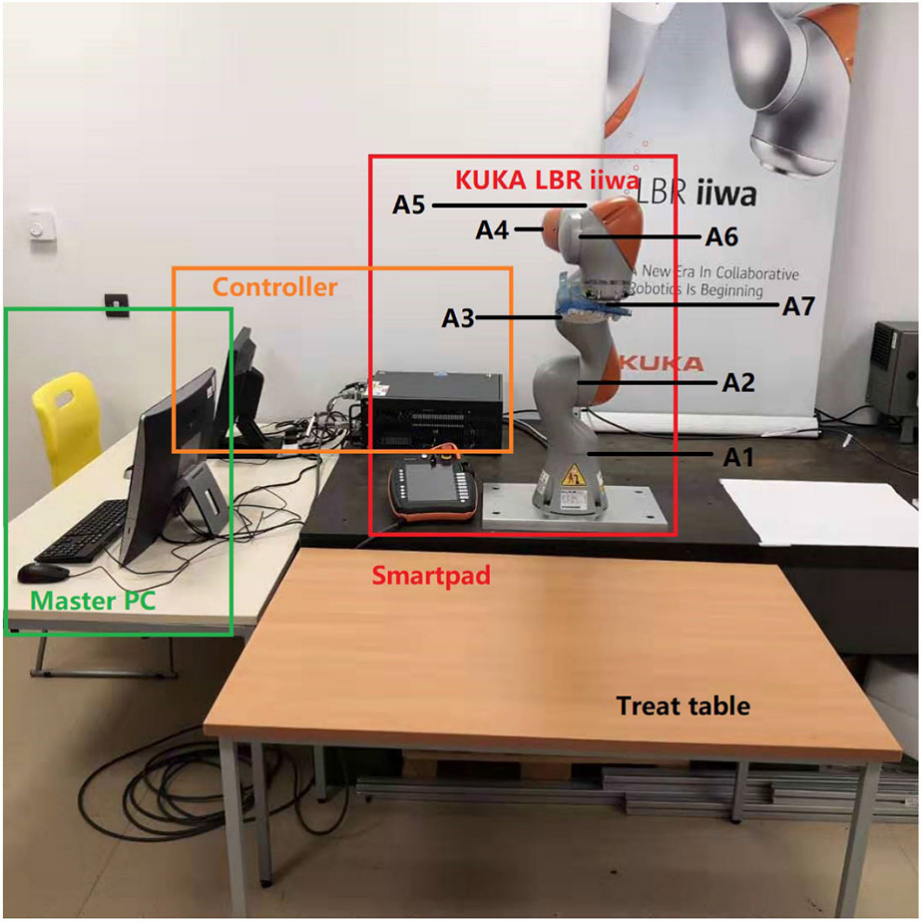
Illustration of the experimental system.

A KUKA LBR iiwa robot, which has 7 DOFs of flexible joints, is implemented in our experimental studies to validate the proposed method. It is controlled utilizing the KUKA Smartpad. A massage glove was attached to the end-effector of the robot. As can be seen from Figure 4, there is a master computer lying by the robotic controller for offline data training purposes, and it is linked to the robot controller by an Ethernet cable. The labels A1–A7 in Figure 4 show the joint actuator's position with force sensor each. In addition, there is a treat table placed in front of the robot base, which is in the workspace of the robot manipulator.

The control frequency is set as 10Hz for the KUKA LBR iiwa manipulator, and the running time is limited to 30 s for the massage path tracking, and thus 300 time samplings are executed for the control loop. The endpoint stiffness in X, Y, and Z directions, Roll, Pitch, and Yaw orientations are set as 1,000, 1,000, 100, 300, 300, and 300 N/m, respectively. The control gains *M*_*g*_, *C*_*g*_ and *K*_*g*_ are gain matrix of the mass, damping and stiffness and respectively set as *diag*[1.0], *diag*[1.0], and 0.5. The reason for setting those values are that we planned the motion path in horizontal plane using GMM-evoluted DMP algorithms with implementing the force in Z directions, which resulted in the impedance effectiveness in the vertical direction of the patient's back.

**Figure 5:**
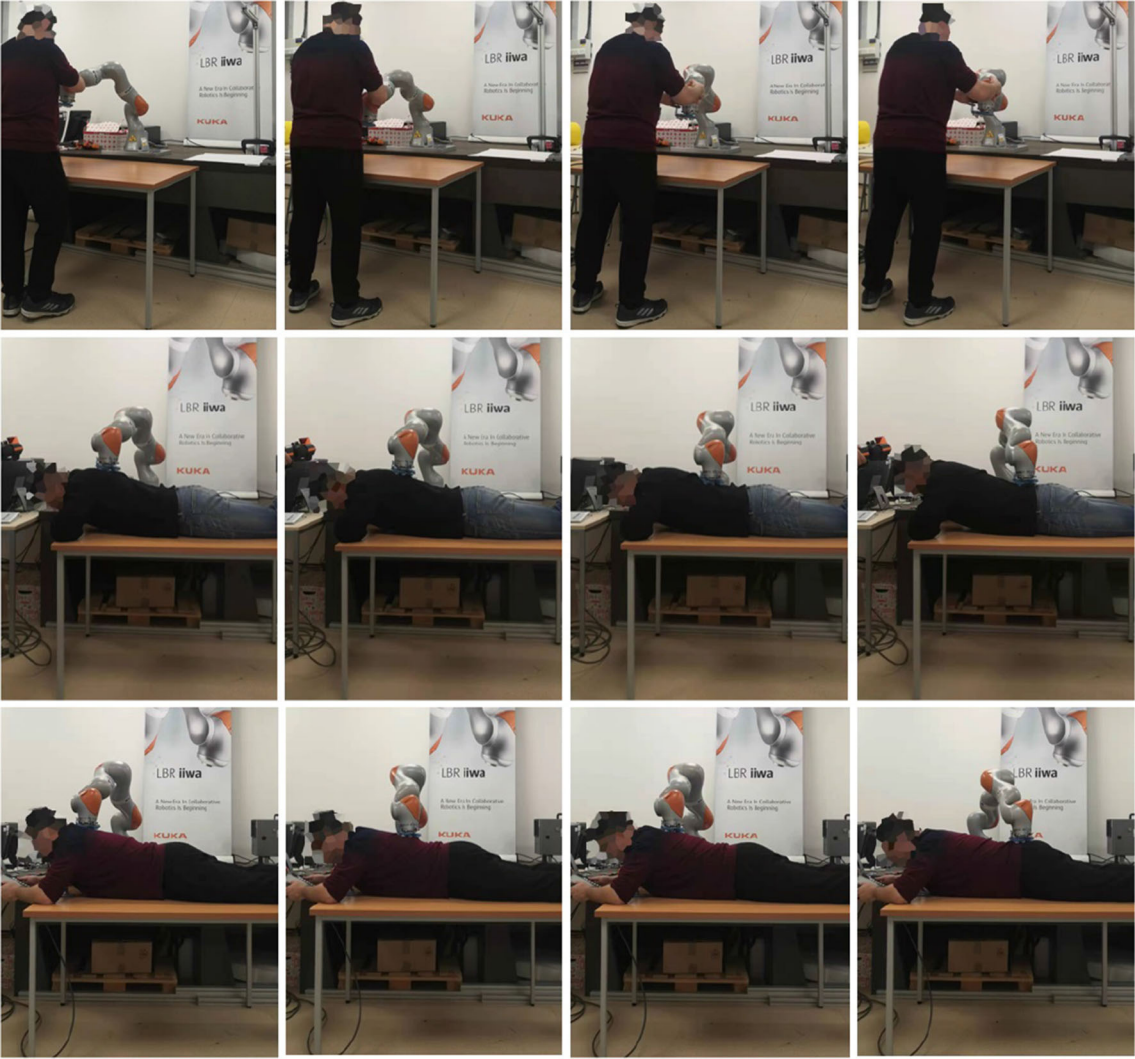
Experiment snapshots of the Kuka LBR iiwa manipulator for massage tasks by the proposed hybrid position/force control method.

First of all, we conducted an experiment to test the accuracy of our proposed robotic teaching interface where only position control was considered. As in some particular situations during the massage, the service robot may require to manipulate as accurately as our human carer; there are specific areas of the patients required to be massaged. This is the reason behind the accuracy of the teaching interface matters. In order to test the accuracy, firstly the position of the KUKA iiwa robot end-effector in the Cartesian space was chosen as the performance index. The human operator physically guided the robot to draw a sine curve for five times in the treat table by holding its end-effector. After training process, the robot could regenerate a new smooth trajectory. For the second experiment, one human operator physically taught the robot to perform the massage movements on the first participant by holding the end-effector of the robot. After the robot was taught, a participant as well as the operator himself were massaged by the robot; the participants slowly and smoothly lifted their back up and down. Figure 5 shows the teaching-based massage process and we can observe that the KUKA LBR iiwa robot successfully accomplished the desired massage task with only one time teaching. Consequently, our proposed massage system could automatically fit different body shapes. Meanwhile, the instantaneous external force and torque of the robot endpoint in Cartesian space were outputted to the master PC for data analysis. The real position in both the Joint space and Cartesian space were plotted by MATLAB.

In addition, the third experiment has been conducted to validate the spatial generalization functions of our proposed robotic teaching interface. To do this, three participants were sought to be undertaken the massage services of their shoulder by seating under the robot manipulator. The operator taught the robot to do the massage task on all the three participants with only once teaching. All the parameters kept the same as those from the second experiment. The massage services were reproduced by the robot for all the participants one by one with different orders.

### 5.2. Experimental Results

**Figure 6:**
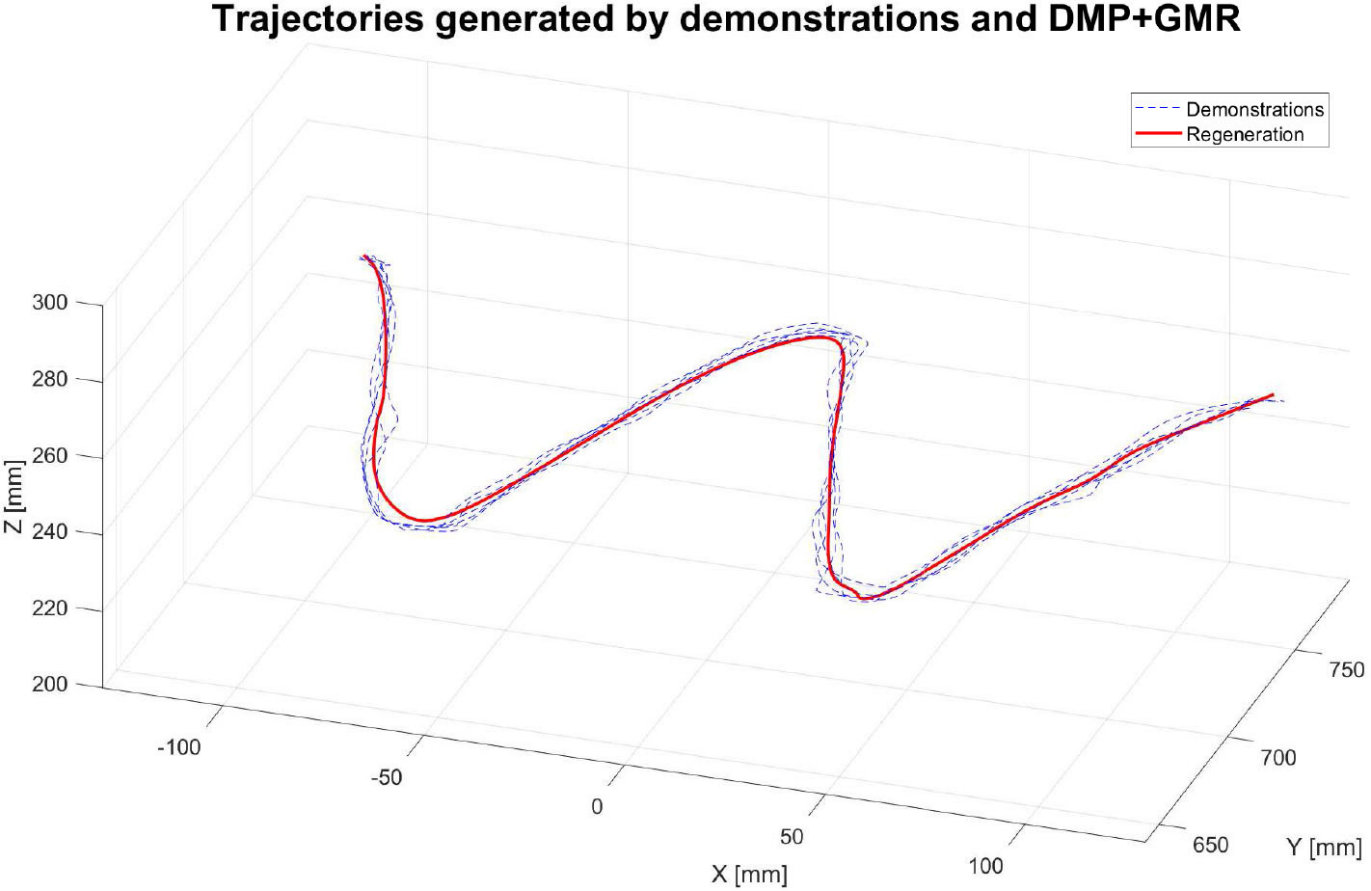
The trajectories of the robot endpoint in Cartesian space generated by demonstrations and the proposed teaching interface.

The first group of experiments aimed to verify the learning performance of our proposed teaching interface when the demonstrations are defective. To verify the learning performance of the modified DMP better, we designed a drawing task for the robot, and the experiment setup is shown in Figure 5. In this experiment, the robot is required to draw an image of sinusoid on the paper after the human operator demonstrates the task five times. The parameters of the DMP model are set as τ = 1, k = 25, c = 10, and α_*f*_ = 8. As is shown in Figure 6, the demonstrations are defective and the curves are irregular. One of the reasons is that the demonstrator is drawing on the paper indirectly by holding the wrist of the robot, which affects the exertion of drawing skill. The demonstrations are modeled in the task space. As is shown in Figure 6, comparing the performance index of the demonstrations and the generated trajectory, a smooth curve is accurately retrieved from multiple demonstrations using the modified DMP without any unexpected drawing. The robot performs the drawing task after learning, and the curve that the robot draws is smoother than the demonstrations.

**Figure 7:**
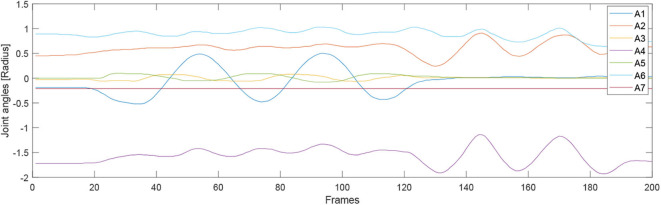
Angular joint values of the robot while massaging the first participant.

**Figure 8:**
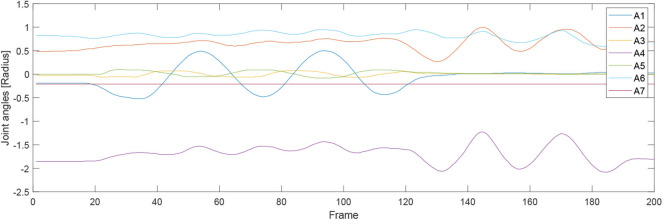
Angular joint values of the robot while massaging the second participant.

**Figure 9:**
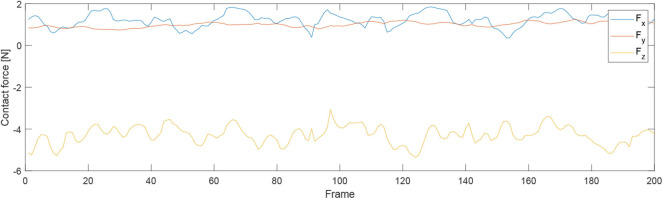
Contact force variables of the end-effector of the robot in X Y Z directions during the massage for the first participant.

**Figure 10:**
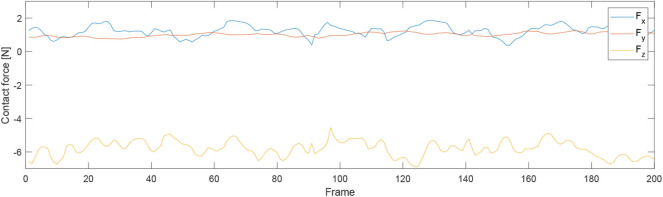
Contact force variables of the end-effector of the robot in X Y Z directions during the massage for the second participant.

**Figure 11:**
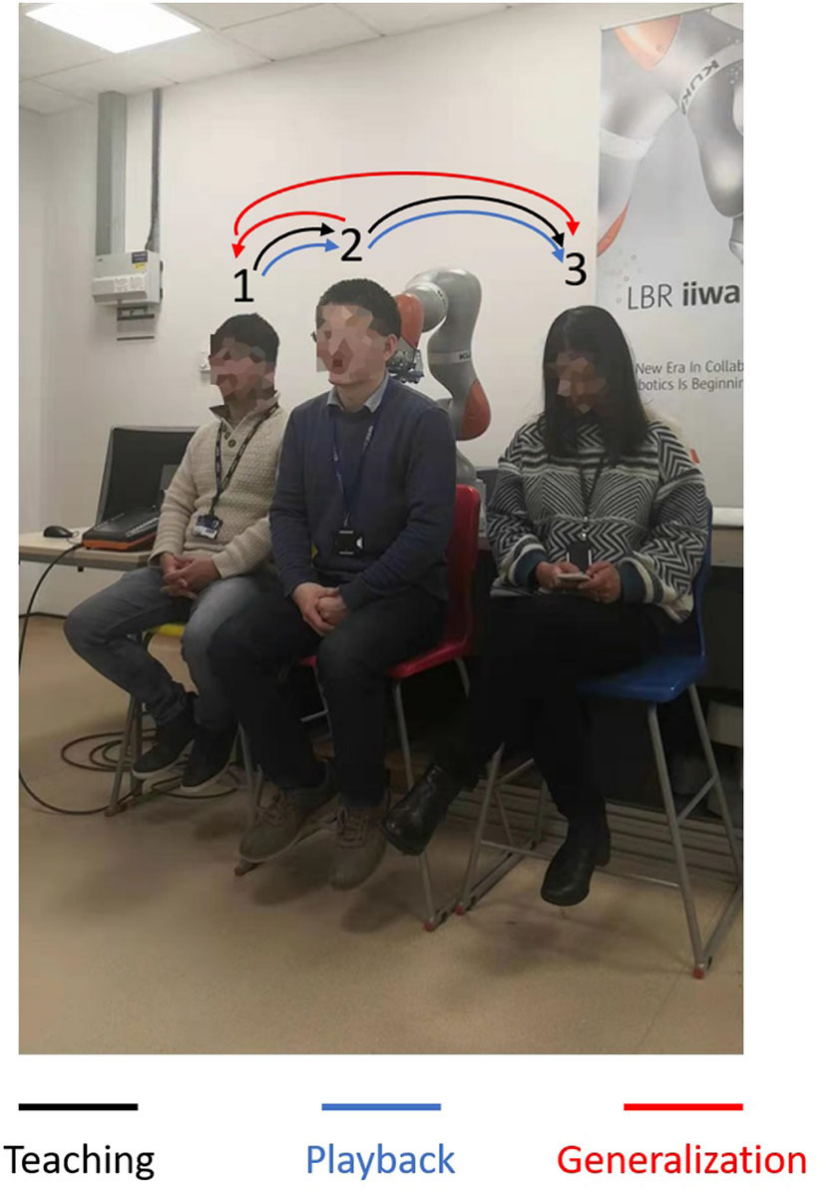
Illustration of the third experimental process.

For the second experiment, two participants are massaged by the robot by only teaching once. During the massaging, the A7 joint of the robot is set as fixed value because it is only related to the end-effector's orientation. Figures 7, 8 illustrate the robot's angular data of all the seven joints when the robot was reproducing the massage on the first and second participant, respectively. Because the massage task was first along the direction of the back of the participants and then pounded the back; from the figures, we can thus see that the first joint of the robot A1 was firstly fluctuating and then kept stable; the A2, A4, and A6 joints were firstly kept stable and then fluctuated. This is because the robot via A1 joint moves left and right; via A2, A4, and A6, joints move up and down. Comparing Figures 7, 8, we can notice that the A2 and A4 joints were increasing, and the A6 joint was decreasing during the whole massage process. This is caused by the fact that the second participant has a thicker body shape, and when the robot is in a lift-up configuration, its 6th joint will be more folded. Figures 9, 10 show the contact force variables of the end-effector of the robot in X, Y, and Z directions during the massage for the first and second participants. Here we define the moving direction is the positive direction of the robot endpoint. We can notice that the contact forces in X and Y directions are the positive values while those in Z direction are negative values with bigger figures. This is caused by the fact that, during the massage paths playback process, the endpoint of the robot in Z direction met resistance comparing to its original teaching configuration. In addition, the contact force vertical to the massage paths (Z direction) also differed with two participants. Contact force variables when massaging the first participant are in the interval [−3.5 −5] N, while the variables when the robot was massaging the second participant are in [−5 −6.5] N. This is owing to the two participants differing in body thickness. The third test has validated the generalization ability of our proposed massaging system. The training results are shown in Figure 11. The motions of the robot are regenerated from an one time teaching based demonstration, which synthesize the features of the demonstration and enable the robot to perform the massage task successfully as shown in Figure 11. The target order of the massage service is then modulated to be changed. A conclusion can be drawn from the video abstract; the profile of the reproduction is obtained.

### 5.3. Remark

Through the above conducted three experiments, we can note several things.

1. Our proposed hybrid position/force mode teaching interface was able to generate an accurate path after being taught, which reduces the errors in 3D space.
2. Our proposed hybrid position/force mode teaching interface was able to automatically and adaptively fit all body shapes with smooth force implementing.
3. The spatial generalization ability was validated, where the whole massage tasks can be segmented into several unit movement primitives, which can be regrouped into different orders with only one-time teaching, and it promoted the working flexibility.

## 6. Conclusion

In this paper, an enhanced force-sensing and robotic-learning algorithm-based robotic teaching interface has been developed to perform the massaging tasks. In the motion generation part, the discrete DMP is selected as the basic motion model, which can generalize the motions. To improve the learning performance of the DMP model, the GMM, and GMR are employed for the estimation of the unknown function of the motion model. With this modification, the DMP model is able to retrieve a better motion from multiple demonstrations of a specific task. For the force input aspects, a hybrid force/position controller is introduced to ensure the safety of direct human-robot interaction. Several experiments have been performed on the KUKA LBR iiwa robot to test the performance of our proposed methods, which has proven that our proposed method can be used to establish a novel robot learning framework for massaging and facilitate the robot learning at a higher level. Our future work will focus on combining with visual monitoring technology, where the acupuncture point and bones of the patient's back will be clearly recognized and tracked, which results in better demonstrations for the robot to learn from.

## Data Availability Statement

All datasets presented in this study are included in the article/Supplementary Material.

## Ethics Statement

The written informed consent was obtained from the individuals for the publication of any potentially identifiable images or data included in this article.

## Author Contributions

CL, AF, JS, and SL: conceptualization. CL and AF: methodology and writing–review and editing. CL: validation and formal analysis. AF and JS: resources. CL and SL: writing–original draft preparation. JS and SL: supervision and funding acquisition. AF: project administration. All authors contributed to the article and approved the submitted version.

## Conflict of Interest

The authors declare that the research was conducted in the absence of any commercial or financial relationships that could be construed as a potential conflict of interest.
